# Effects of *Toona sinensis* Leaf Extract and Its Chemical Constituents on Xanthine Oxidase Activity and Serum Uric Acid Levels in Potassium Oxonate-Induced Hyperuricemic Rats

**DOI:** 10.3390/molecules23123254

**Published:** 2018-12-09

**Authors:** Heung Joo Yuk, Young-Sil Lee, Hyung Won Ryu, Seung-Hyung Kim, Dong-Seon Kim

**Affiliations:** 1 Herbal Medicine Research Division, Korea Institute of Oriental Medicine (KIOM), Daejeon 34054, Korea; yukhj@kiom.re.kr (H.J.Y.); rheeys04@kiom.re.kr (Y.-S.L.); 2 Natural Medicine Research Center, Korea Research Institute of Bioscience and Biotechnology (KRIBB), Cheongju, Chungbuk 28116, Korea; ryuhw@kribb.re.kr; 3 Institute of Traditional Medicine and Bioscience, Daejeon University, Daejeon 34520, Korea; sksh518@dju.kr

**Keywords:** *Toona sinensis*, xanthine oxidase, uric acid, hyperuricemia, ultraperformance liquid chromatography-quadrupole time-of-flight mass spectrometry

## Abstract

*Toona sinensis* leaf is used as a seasonal vegetable in Korea. A 70% ethanol extract of these leaves exhibited potent xanthine oxidase (XO) inhibition, with a 50% inhibitory concentration (IC_50_) of 78.4 µM. To investigate the compounds responsible for this effect, bioassay-guided purification led to the isolation of five constituents, identified as quercetin-3-*O*-rutinoside, quercetin-3-*O*-β-d-glucopyranoside, 1,2,3,4,6-penta-*O*-galloyl-β-d-glucopyranose (compound **3**), quercetin-3-*O*-α-l-rhamnopyranoside, and kaempferol-3-*O*-α-l-rhamnopyranoside. Compound **3** showed the most potent inhibition of XO, with an IC_50_ of 2.8 µM. This was similar to that of allopurinol (IC_50_ = 2.3 µM), which is used clinically to treat hyperuricemia. Kinetic analyses found that compound **3** was a reversible noncompetitive XO inhibitor. In vivo, the *T. sinensis* leaf extract (300 mg/kg), or compound **3** (40 mg/kg), significantly decreased serum uric acid levels in rats with potassium oxonate-induced hyperuricemia. Furthermore, ultraperformance liquid chromatography-quadrupole time-of-flight mass spectrometry analysis identified a high level of compound **3** in the leaf extract. These findings suggest that *T. sinensis* leaves could be developed to produce nutraceutical preparations.

## 1. Introduction

Hyperuricemia results from overproduction or underexcretion of uric acid [1,2] and is characterized by high serum uric acid levels (>7 mg/dL in humans). This increases the precipitation of urate crystals in the joints and kidneys, causing gout and gouty arthritis [3]. Recent publications have shown that serum uric acid levels are associated with diseases such as metabolic syndrome, hypertension, and increased cardiovascular risk [4,5]. Xanthine oxidase (XO) is an important enzyme that catalyzes the oxidation of hypoxanthine to xanthine and the subsequent oxidation of xanthine to uric acid (urate) in humans [6]. XO inhibitors such as allopurinol and febuxostat reduce uric acid synthesis and act as useful clinical treatments for hyperuricemia and gout [7,8]. However, these synthetic drugs also produce side effects such as skin rash, renal failure, renal toxicity, and allergic reactions. It would therefore be beneficial to develop alternative drugs with increased efficacy and fewer side effects.

*Toona sinensis* M.Roem. (also known as *Cedrela sinensis* in Asia) is a member of the Meliaceae family, which is widely distributed throughout the world. The young leaves of *T. sinensis* can be eaten as a seasonal vegetable. In addition, most parts of this plant, including the stem bark, root bark, and young shoots, have been used as medicinal resources in eastern Asia [9,10]. Previous studies have revealed that aqueous leaf extracts were protective against hydrogen peroxide-induced oxidative stress and DNA damage in Madin–Darby canine kidney cells [11]. Recently, a *T. sinensis* extract was also shown to enhance anti-influenza A (H1N1) viral effects via significant downregulation of adhesion chemokines [12]. Additionally, *T. sinensis* leaves are a well-known source of flavonoids, phenolic acids, retinoids, triterpenes, and vitamins: These compounds have potential health-promoting properties related to their antioxidant, anticancer, antidiabetes, and anti-inflammatory effects [13,14]. Many researchers have reported that compounds isolated from *T. sinensis* leaves contributed to their biological activities, but no detailed investigation of potential XO inhibitors has been carried out.

In the present study, we identified XO inhibitory properties and isolated five compounds (**1**–**5**) from *T. sinensis* leaves. Kinetic studies indicated that compound **3** exhibited the most potent inhibitory activity. We also studied the effects of the most abundant compound, (**4**), and the most potent XO inhibitor, (**3**), on serum uric acid levels in rats with potassium oxonate (PO)-induced hyperuricemia. In addition, our quantification of the compounds isolated from *T. sinensis* could inform quality assessment of *T. sinensis* leaf extracts.

## 2. Results and Discussion

### 2.1. Bioassay-Guided Isolation and Structural Identification

**Figure 1:**
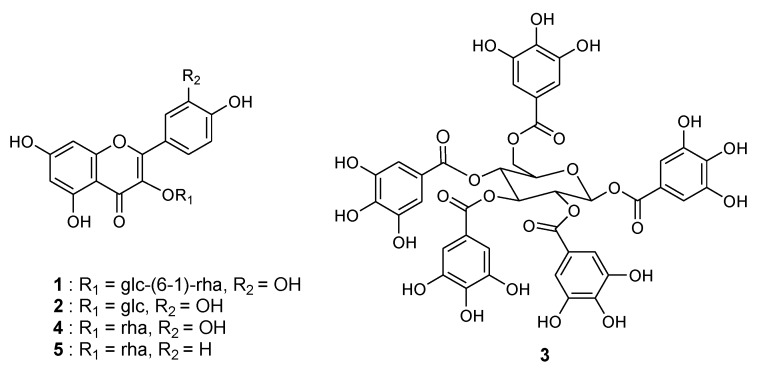
Chemical structures of isolated compounds (**1**−**5**) from the leaves of *T. sinensis*.

Extracts of *T. sinensis* leaves were prepared using different polar solvents (ethyl acetate [EtOAc], 70% ethanol in water [70% EtOH], H_2_O) and tested for XO inhibitory activity. Enzyme activity was assayed spectrophotometrically by determining uric acid formation at 295 nm, using xanthine as the substrate [15]. As shown in Table 1, the 70% EtOH extract of *T. sinensis* leaves (TSE) exhibited potent XO inhibition, with a 50% inhibitory concentration (IC_50_) of 78.4 μg/mL. We then investigated which active compounds were responsible for this effect. Activity-guided fractionation of TSE gave five compounds, which were purified using Diaion, octadecyl-functionalized silica gel, and Sephadex LH-20 column chromatography. The isolated compounds (**1**–**5**) were identified as quercetin-3-*O*-rutinoside (rutin, **1**) [16], quercetin-3-*O*-β-d-glucopyranoside (isoquercitrin, **2**) [17], 1,2,3,4,6-penta-*O*-galloyl-β-d-glucopyranose (pentagalloyl glucose, **3**) [18], quercetin-3-*O*-α-l-rhamnopyranoside (quercetrin, **4**) [19], and kaempferol-3-*O*-α-l-rhamnopyranoside (afzelin, **5**) [20], through analysis of our spectroscopic data (^1^H- and ^13^C-nuclear magnetic resonance (NMR) and high resolution electrospray ionization (HRESI)-MS, Supplementary Materials) and by comparison to previous literature. The structures of the compounds isolated from *T. sinensis* leaves are shown in Figure 1. 

Pentagalloyl glucose (**3**), the precursor of gallotannin, can be found in several medicinal plants [21], and this was the most effective XO inhibitor. Compound **3** was obtained as a white power with a molecular formula of C_41_H_32_O_26_ and 26 degrees of unsaturation, established by HRESI-MS ([M − H]^−^ at *m/z* 939.1119, calcd for C_41_H_31_O_26_ 939.1104, 1.6 ppm error). The ^13^C-NMR data identified 15 C–C double bonds and five carbonyl groups, thus accounting for 20 of the 26 degrees of unsaturation. The remaining 6 degrees of unsaturation were ascribed to five aromatic rings and one sugar ring. In MS fragmentation analysis, compound **3** showed fragment ions at *m/z* 787, 769, 617, 465, 313, and 169 in the MS^2^ spectrum. This fragmentation pattern was consistent with previous reports [22]. Thus, compound **3** was identified as 1,2,3,4,6-penta-*O*-galloyl-β-d-glucopyranose.

### 2.2. Contribution of the Identified Compounds to XO Inhibitory Activity

**Figure 2:**
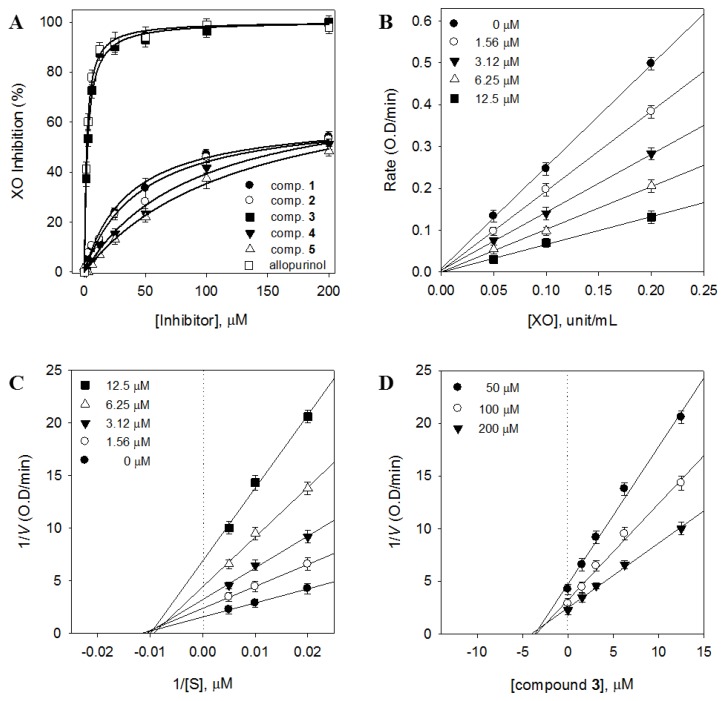
(**A**) Inhibitory effects of compounds (**1**−**5**) on the activity of xanthine oxidase (XO) for the oxidation of xanthine to uric acid. (**B**) Catalytic activity of XO as a function of enzyme concentration at different concentrations of compound **3**. (**C**) Lineweaver–Burk plots were constructed for the inhibition of XO by compound **3**. The plot is expressed as 1/velocity versus 1/xanthine (S) with or without an inhibitor in the reaction solutions. (**D**) Dixon plots of XO inhibition by compound **3**. The graphical symbols are substrate concentrations (50 μM, ●; 100 μM, ○; 200 μM, ▼).

Compounds **1**–**5** were evaluated for their inhibitory effects on XO activity. Both flavonol glycosides (**1**, **2**, **4**, and **5**) and gallotannin (**3**) had concentration-dependent effects on XO activity (Figure 2A). Cos et al. reported that flavonoids such as quercetin and kaempferol had strong XO inhibitory effects [23]. However, our results showed that the flavonoid compounds (**1**, **2**, **4**, and **5**) were inactive (IC_50_ > 100 μM). These data were striking because this class of XO inhibitor did not behave in accordance with the usual trend, where glycosides usually show significantly less inhibitory potency than their aglycone counterpart. Among the five compounds, pentagalloyl glucose (**3**) exhibited the most potent inhibitory activity (96.4%) at a concentration of 100 μM, followed by compounds **2** (47.8%), **1** (42.9%), **4** (38.9%), and **5** (31.7%). The potency of compound **3** (IC_50_ = 2.79 μM) could be favorably compared to selective XO inhibitors currently used as therapeutics, including allopurinol (IC_50_ = 2.28 μM) [24]. It is known that allopurinol and oxypurinol (isosteres of hypoxanthine and xanthine, respectively) inhibit xanthine oxidase activity and limit the biosynthesis of uric acid. However, our results showed that oxypurinol (IC_50_ = 28.4 µM) was not as effective in inhibiting the activity of XO when compared to allopurinol. In regard to the affinity of XO for oxypurinol, Tamta et al. have also reported that oxypurinol showed a much lower inhibitory activity than allopurinol in XO screening [25]. The binding of oxypurinol to the reduced molybdenum site of the enzyme has been shown to be reversible and is released after reoxidation of the enzyme. Although the inhibitor binds very tightly to the enzyme, the inhibition is time dependent and requires some time for complete enzyme inhibition [26].

As shown in Figure 2B, the effects of various concentrations of **3** (0, 1.56, 3.12, 6.25, and 12.5 μM) on the activity of different XO concentrations (0, 0.05, 0.1, and 0.2 unit/mL) were analyzed. These data indicated that compound **3** acted as a reversible XO inhibitor. We subsequently analyzed the type of inhibition using both Lineweaver–Burk (Figure 2C) and Dixon (Figure 2D) plots. These analyses revealed that the 1/*y*-intercept (*V*_max_) decreased, whereas the −1/*x*-intercept (*K*_m_) remained constant, as the compound concentration increased. This indicated that compound **3** acted as a noncompetitive inhibitor with an inhibition constant (*K*_i_) value of 3.1 μM. 

### 2.3. Serum Uric Acid Levels in Rats with PO-Induced Hyperuricemia

**Figure 3:**
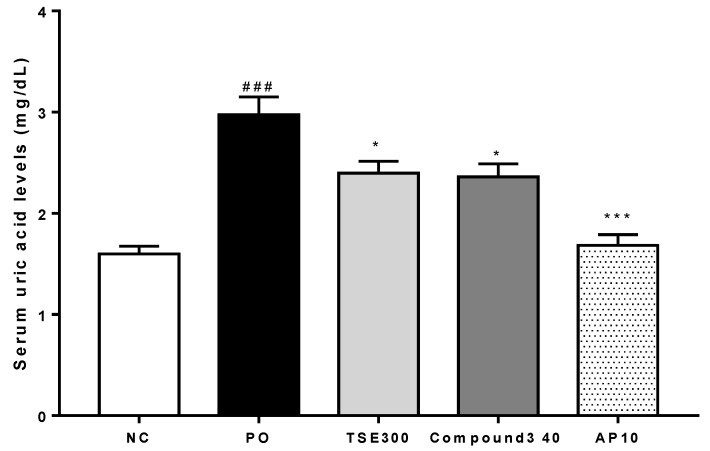
Effects of 70% EtOH *T. sinensis* leaves (TSE) and compound **3** on serum uric acid levels in potassium oxonate (PO)-induced hyperuricemic rats. NC: normal control group; PO: potassium oxonate-induced hyperuricemia group; TSE-300: 300 mg/kg 70% EtOH TSE; AP-10: 10 mg/kg allopurinol. Data are expressed as the mean ± SEM (*n* = 6); ^###^ *p* < 0.001 versus the NC group; * *p* < 0.05 and *** p < 0.005 versus the PO group.

The selective uricase inhibitor, PO, is known to produce hyperuricemia by blocking the effects of hepatic uricase [27]. The effects of TSE and compound **3** on PO-induced hyperuricemic rats are shown in Figure 3. Serum uric acid levels were significantly increased in the PO-treated control rats, as compared to the normal control group (*p* < 0.005). The rats treated with TSE (300 mg/kg) or compound **3** (40 mg/kg) showed significant (19.4% and 20.7%) reductions in their serum uric acid levels, as compared to the PO group (both *p* < 0.01). 

Rats treated with allopurinol (10 mg/kg) as a positive control showed a 43.5% decrease in their serum uric acid level (*p* < 0.005). Although compound **3** had a potent inhibitory effect on XO activity in vitro (IC_50_ = 2.79 μM), it did not reduce serum uric acid levels as effectively as allopurinol in vivo. The most abundant *T. sinensis* compound, **4,** had no effect on the serum uric acid level at 40 mg/kg (Supplementary Materials). Discrepancies between the in vitro and in vivo results may have reflected differences in bioavailability and pharmacokinetics in rats. It is also possible that structural differences, together with variations in absorption and metabolic characteristics of the various test compounds, may have led to the formation of metabolites with contrasting XO inhibitory activity.

### 2.4. Ultraperformance Liquid Chromatography-Quadrupole Time-of-Flight Mass Spectrometry (UPLC-qToF MS) Profiles

**Figure 4:**
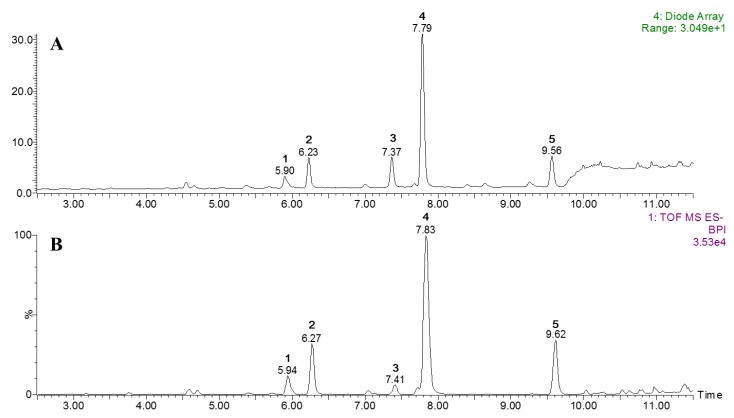
Representative chromatograms of 70% EtOH extract from the leaves of *T. sinensis*: (**A**) Photodiode array (PDA) chromatogram and (**B**) total ion current-base peak intensity (TIC-BPI) chromatogram.

The constituents of TSE were analyzed using UPLC-qToF MS. As shown in Figure 4, complete chromatographic separation of these constituents was achieved within 13 min. The identity of each peak in the UPLC trace was doubly verified by comparison to the retention time and the UV spectrum (*λ*_max_) of the isolated pure compound. All detected peaks also showed molecular ions with masses consistent with the identified compounds (**1**, [M − H]^−^ at *m/z* 609.1448; **2**, *m/z* 463.0893; **3**, *m/z* 939.1119; **4**, *m/z* 447.0939; **5**, *m/z* 431.0974), as shown in Table 2. Extractions for quantitative analyses were performed using EtOAc, 70% EtOH, or H_2_O. The most abundant peaks (**1**–**5**) were observed using 70% EtOH. The levels of the five isolated compounds were analyzed by UPLC at 254 nm for the flavonoids (**1**, **2**, **4**, and **5**) and at 280 nm for the gallotannin (**3**). The standard curves were linear and reproducible for each of the isolated compounds, as evidenced by the correlation coefficients (*r*^2^ = 0.998–0.999). The levels of compounds **1**–**5** in TSE were determined as 0.69, 1.32, 1.34, 7.12, and 1.46 mg/g of dried sample, respectively. In a preliminary study, the extracts produced using EtOAc or H_2_O showed weak inhibitory effects on XO (IC_50_ values of >500 μg/mL and 284.7 μg/mL, respectively), and the levels of compounds **1**–**5** were also significantly lower than those present in 70% EtOH TSE.

## 3. Materials and Methods 

### 3.1. Plant Materials and Sample Preparation

*T. sinensis* leaves were collected at tree communities in Sacheon, South Korea, in May 2016. The collected leaves were identified, and a voucher specimen (KRIBB 0000583) was deposited in the Korea Research Institute of Bioscience and Biotechnology. The collected leaves were freeze-dried immediately after sampling and then ground to a powder and stored at −75 °C until further analysis. Three different solvent extraction systems were used: EtOAc, 70% EtOH, and distilled H_2_O. All samples were sonicated twice for 30 min at 25 °C, and aliquots were filtered through a 0.2 μm PTFE filter prior to use in enzymatic assays and liquid chromatography analyses. All of the extraction and chromatographic solvents employed were of LC–MS grade (J. T. Baker, Phillipsburg, NJ, USA).

### 3.2. Instruments

^1^H and ^13^C NMR experiments were performed on a Bruker AM 400, using different solvents (CD_3_OD and DMSO-*d*_6_). The internal standard was tetramethylsilane (TMS) (Andover, MA, USA). HRESI mass spectra were obtained on a qToF mass spectrometer (qToF Premier^TM^, Waters Corp., Milford, MA, USA). The UPLC system, equipped with a binary solvent delivery system, an autosampler, and a UV detector, was also from Waters Corp. Separations were carried out on a medium pressure liquid chromatography (MPLC) system (LC-forte/R; YMC Co., Ltd., Kyoto, Japan), where reversed-phase (RP) cartridges purchased from YMC were employed. For purification, preparative high-performance liquid chromatography (HPLC) was performed using a Gilson HPLC system (Gilson, Inc., Middleton, WI, USA) with a YMC octadecyl-functionalized silica gel-AQ column (250 mm × 20 mm, i.d. 5 μm). A Multi-Mode Microplate Reader SpectraMax M2 (Molecular Devices, Sunnyvale, CA, USA) was used for the enzymatic assays.

### 3.3. Sample Extraction, Fractionation, and Isolation

The freeze-dried *T. sinensis* leaves (200 g) were cut into small pieces and extracted with 70% ethanol (4 L × 3) at room temperature. The combined extract was evaporated, resulting in 36.4 g (18.2% yield) of crude extract. A 20 g sample of this extract was subjected to column chromatography on a Diaion HP20SS (10 × 40 cm, 750 g), eluted with MeOH/H_2_O mixtures [0:100 (1 L), 30:70 (1 L), 50:50 (2 L), 70:30 (2 L), 90:10 (1 L), and 100:0 (1 L)], to give six fractions (A–F). Fraction C (2.3 g) was fractionated on an RP column (120 g, C18 cartridge) using MPLC with a linear gradient of 30%–90% MeOH/H_2_O at a flow rate of 20 mL/min to afford eight fractions (C1–C8). Subfractions C3–C5, enriched with compounds **1** and **2**, were combined (342 mg) and further purified by Sephadex LH-20 with 70% MeOH as the eluent, yielding compounds **1** (49 mg) and **2** (112 mg). Fraction D (4.5 g) was fractionated via RP-MPLC using a C18 column cartridge (220 g) using a gradient of increasing MeOH (50%–90%) in H_2_O to give fractions D1–D11. Subfractions D4–C7, enriched with compounds **3** and **4**, were combined (941 mg) and further purified by preparative HPLC using 60% MeOH as the mobile phase to afford compounds **3** (114 mg) and **4** (623 mg). Subsequent separation of fraction D9 (230 mg), enriched with compound **5**, on Sephadex LH-20 with 80% MeOH as eluent yielded compound **5** (126 mg). The physicochemical and spectrometric data of the five compounds (**1**–**5**) were as follows:

*Quercetin-3-O-rutinoside* (**1**). Yellow powder; negative ESI-HRMS, *m/z*: 609.1448 [M − H]^−^ (calcd for C_27_H_29_O_16_ 609.1456); ^1^H NMR (400 MHz, DMSO-*d*_6_): *δ* 7.56 (1H, d, *J* = 2.1 Hz, H-2′), 7.53 (1H, dd, *J* = 2.1 Hz, 8.1 Hz, H-6′), 6.84 (1H, d, *J* = 8.2 Hz, H-5′), 6.38 (1H, d, *J* = 1.9 Hz, H-8), 6.19 (1H, d, *J* = 1.9 Hz, H-6), 5.34 (1H, d, *J* = 7.2 Hz, H-1′′), 4.39 (s, H-1′′′), 3.70 (2H, d, *J* = 9.7 Hz, H-6′′), 1.10 (3H, d, *J* = 6.1 Hz, CH_3_-6′′′); ^13^C NMR (100 MHz, DMSO-*d*_6_): *δ* 178.1 (s, C-4), 166.5 (s, C-7), 163.4 (s, C-5), 158.8 (s, C-9), 158.7 (s, C-2), 147.0 (s, C-4′), 144.5 (s, C-3′), 135.6 (s, C-3), 123.8 (d, C-6′), 123.4 (s, C-1′), 118.5 (d, C-5′), 117.5 (d, C-2′), 106.2 (s, C-10), 103.5 (d, C-1′′), 103.0 (d, C-1′′′), 100.9 (d, C-6), 95.9 (d, C-8), 78.7 (d, C-3′′), 78.1 (d, C-5′′), 76.3 (d, C-2′′), 74.1 (d, C-4′′′), 72.8 (d, C-3′′′), 72.6 (d, C-2′′′), 70.1 (d, C-4′′), 72.3 (d, C-5′′′), 70.5 (t, C-6′′), 20.0 (q, C-6′′′).

*Quercetin-3-O-β-d-glucopyranoside* (**2**). Yellow powder; negative ESI-HRMS, *m/z*: 463.0893 [M − H]^−^ (calcd for C_21_H_19_O_12_ 463.0877); ^1^H NMR (400 MHz, DMSO-*d*_6_): *δ* 7.59 (1H, d, *J* = 2.0 Hz, H-2′), 7.58 (1H, dd, *J* = 2.0, 8.8 Hz, H-6′), 6.86 (1H, d, *J* = 8.8 Hz, H-5′), 6.40 (1H, d, *J* = 2.0 Hz, H-8), 6.20 (1H, d, *J* = 2.0 Hz, H-6), 5.47 (1H, d, *J* = 7.4 Hz, H-1′′), 3.60–3.09 (m, glc protons); ^13^C-NMR (DMSO-*d*_6_, 100 MHz) *δ* 177.7 (s, C-4), 164.7 (s, C-7), 161.6 (s, C-5), 156.7 (s, C-9), 156.5 (s, C-2), 148.8 (s, C-4′), 145.2 (s, C-3′), 133.7 (s, C-3), 121.9 (s, C-1′), 121.5 (d, C-6′), 116.5 (d, C-2′), 115.6 (d, C-5′), 104.3 (s, C-10), 101.2 (d, C-1′′), 99.1 (d, C-6), 93.9 (d, C-8), 77.9 (d, C-5′′), 76.9 (d, C-3′′), 74.4 (d, C-2′′), 70.3 (d, C-4′′), 61.3 (t, C-6′′).

*1,2,3,4,6-penta-O-galloyl-β-d-glucopyranose* (**3**). White powder; negative ESI-HRMS, *m/z*: 939.1119 [M − H]^−^ (calcd for C_41_H_31_O_26_ 939.1104); ^1^H NMR (400 MHz, CD_3_OD): *δ* 7.13, 7.07, 6.99, 6.97, 6.92 (each 2H, s, galloyl H-2, 6), 6.26 (1H, d, *J* = 8.4 Hz, H-1′), 5.93 (1H, t, *J* = 9.5, 9.6 Hz, H-3′), 5.65 (1H, t, *J* = 8.7, 9.5 Hz, H-4′), 5.60 (1H, dd, *J* = 8.5, 9.6 Hz, H-2′), 4.53 (1H, d, *J* = 10.8 Hz, H-5′), 4.38–4.46 (2H, m, H-6′); ^13^C NMR (100 MHz, CD_3_OD): *δ* 168.3, 167.7, 167.4, 167.3, 166.6 (each s, C-7), 147.0, 146.9, 146.8, 146.7, 146.6 (each s, C-3, 5), 141.2, 140.8, 140.7, 140.5, 140.4 (each s, C-4), 121.4, 120.7, 120.6, 120.5, 120.1 (each s, C-1), 110.9, 110.8, 110.7, 110.7, 110.6 (each d, C-2, 6), 94.2 (d, C-1′), 74.8 (d, C-5′), 74.5 (d, C-3′), 72.6 (d, C-2′), 70.1 (d, C-4′), 63.5 (t, C-6′).

*Quercetin-3-O-α-l-rhamnopyranoside* (**4**). Yellow powder; negative ESI-HRMS, *m/z*: 447.0939 [M − H]^−^ (calcd for C_21_H_19_O_11_ 447.0927); ^1^H NMR (400 MHz, DMSO-*d*_6_): *δ* 7.30 (1H, d, *J* = 2.0 Hz, H-2′), 7.26 (1H, dd, *J* = 2.0, 8.8 Hz, H-6′), 6.87 (1H, d, *J* = 8.4 Hz, H-5′), 6.39 (1H, d, *J* = 1.2 Hz, H-8), 6.20 (1H, d, *J* = 1.2 Hz, H-6), 5.26 (brs, H-1′′), 3.98 (brs, H-2′′), 3.52 (1H, dd, *J* = 8.8, 8.8 Hz, H-3′′), 3.22 (m, H-5′′), 3.14 (m, H-4′′), 0.82 (3H, d, *J* = 6.0 Hz, H-6′′); ^13^C-NMR (DMSO-*d*_6_, 100 MHz) *δ* 178.1 (s, C-4), 164.7 (s, C-7), 161.6 (s, C-5), 157.6 (s, C-9), 156.8 (s, C-2), 148.8 (s, C-4′), 145.6 (s, C-3′), 134.6 (s, C-3), 121.5 (s, C-1′), 121.1 (d, C-6′), 115.9 (d, C-5′), 115.8 (d, C-2′), 104.4 (s, C-10), 102.2 (d, C-1′′), 99.1 (d, C-6), 94.0 (d, C-8), 71.5 (d, C-4′′), 70.9 (d, C-3′′), 70.7 (d, C-2′′), 70.4 (d, C-5′′), 17.9 (q, C-6′′).

*Kaempferol-3-O-α-l-rhamnopyranoside* (**5**). Yellow powder; negative ESI-HRMS, *m/z*: 431.0974 [M − H]^−^ (calcd for C_21_H_19_O_10_ 431.0978); ^1^H NMR (400 MHz, DMSO-*d*_6_): *δ* 7.76 (2H, d, *J* = 8.8 Hz, H-2′, 6′), 6.92 (2H, d, *J* = 8.8 Hz, H-3′, 5′), 6.42 (1H, d, *J* = 2.0 Hz, H-8), 6.21 (1H, d, *J* = 2.0 Hz, H-6), 5.30 (1H, br s, H-1′′), 3.99 (1H, br s, H-2′′), 3.48 (1H, d, *J* = 6.4 Hz, H-3′′), 3.15–3.07 (2H, m, H-4′′, 5′′), 0.80 (3H, d, *J* = 6.0 Hz, H-6′′); ^13^C-NMR (DMSO-*d*_6_, 100 MHz) *δ* 178.1 (C-4), 164.6 (C-7), 161.6 (C-5), 160.3 (C-4′), 157.6 (C-9), 156.9 (C-2), 134.6 (C-3), 130.9 (C-2′, 6′), 120.9 (C-1′), 115.7 (C-3′, 5′), 104.5 (C-10), 102.1 (C-1′′), 99.1 (C-6), 94.1 (C-8), 71.5 (C-4′′), 70.9 (C-2′′), 70.7 (C-3′′), 70.4 (C-5′′), 17.8 (C-6′′).

### 3.4. UPLC-qToF MS Analysis

The identification of component peaks was carried out by UPLC using a photodiode array detector. Test sample aliquots (2.0 μL) were then injected into a C18 BEH column (2.1 × 100 mm, 1.7 μm) at a flow rate of 0.4 mL/min. The mobile phase consisted of water/acetonitrile (ACN) containing 0.1% formic acid. The linear gradient was as follows: 0–1 min, 10% ACN; 1–10 min, 10–40% ACN; 10–11.3 min, 40–100% ACN; 11.3–13.3 min, 100% ACN; 13.3–15 min, return to 10% ACN. The qToF MS was operated in negative-ion mode under the following conditions: Source temperature, 110 °C; cone voltage, 45 V; capillary voltage, 2.5 kV. The full-scan data and MS/MS spectra were collected using MassLynx software (Waters Corp.).

### 3.5. In Vitro XO Assay

XO activity was assayed spectrophotometrically on a Spectra Max M2 microplate reader, using published experimental procedures, with slight modifications. First, 135 µL of 100 mM sodium pyrophosphate buffer (HCl, pH 7.5), 20 µL of 0.2 unit XO enzyme (bovine milk) in buffer, and 5 µL of sample (test extract or compound) in DMSO were mixed at 37 °C. The reaction was started by adding 40 µL of substrate (0.5 mM xanthine) in buffer to the mixture. The reaction mixture (200 µL) was incubated at 37 °C in a 300 µL well plate, and the UV absorbance was determined at 295 nm. Allopurinol, a known inhibitor of XO, served as a positive control.

### 3.6. Induction of Hyperuricemia and Sample Treatment

Male Sprague–Dawley rats (7 weeks old) were purchased from Orient Bio (Seongmam, Korea) and were housed at a temperature of 22 ± 1 °C with 50 ± 10% humidity under a 12 h light/dark cycle with free access to a laboratory diet and water. The experimental design was approved by the Committee on Animal Care of the Korea Institute of Oriental Medicine, and all experiments were performed in accordance with committee guidelines. The uricase inhibitor PO was administered to induce hyperuricemia. The rats were divided into the following five groups (*n* = 6/group): (1) Normal control group; (2) PO-treated control group; (3) PO + 300 mg/kg TSE group; (4) PO + 40 mg/kg compound **3** group; (5) PO + 10 mg/kg allopurinol group. The rats in groups 2–5 were injected intraperitoneally with 150 mg/kg PO prepared in 0.5% CMC with 0.1 M sodium acetate (pH 5.0) to induce hyperuricemia, and the normal control rats were treated with 0.5% CMC with 0.1 M sodium acetate. TSE, compound **3**, and allopurinol were dispersed in 0.5% CMC and administered by oral gavage 1 h after PO injection. 

### 3.7. Serum Uric Acid Analysis

Blood samples were collected via cardiac puncture under anesthesia 2 h after oral treatment. Serum was obtained by centrifugation of blood at 3000× *g* for 10 min at 4 °C. The separated serum was stored at −80 °C until analysis. Serum uric acid levels were determined by an enzymatic-colorimetric method using a commercial assay kit (Biovision, Milpitas, CA, USA) according to the manufacturer’s protocol.

### 3.8. Statistical Analysis

All measurements of in vitro XO activity and individual compound levels were conducted in triplicate. Data are expressed as the mean ± standard deviation using Sigma plot 10.0 software (Systat Software Inc., San Jose, CA, USA). In vivo data are expressed as the mean ± standard error of the mean. Differences between the control and treatment groups were analyzed by one-way analysis of variance, Dunnett’s multiple comparison test was applied to determine significance using Prism 7.0 (GraphPad Software Inc., San Diego, CA, USA), and *p* < 0.05 was considered statistically significant.

## 4. Conclusions

TSE had a significant inhibitory effect on XO in vitro and produced a significant hypouricemic effect on rats with PO-induced hyperuricemia in vivo. Importantly, the main active component of TSE was found to be pentagalloyl glucose (**3**), which showed strong inhibition (IC_50_ = 2.79 μM) of XO and also lowered serum uric acid levels in rats with PO-induced hyperuricemia. Based on our detailed kinetic analyses using double-reciprocal plots, compound **3** was a noncompetitive inhibitor with a *K*_i_ of 3.1 μM. Compound **3** was present at a level of 1.34 mg/g of dried TSE. These findings suggest that *T. sinensis* leaves could be developed to produce health foods and nutraceuticals.

## Figures and Tables

**Table 1:** Inhibitory effects of the leaf extract of *Toona sinensis* using different solvents and isolated compounds **1**−**5** on xanthine oxidase activities.

Compound	Xanthine Oxidase		
	IC_50_^a^	Inhibition % ^b^	Kinetic Mode (*K*_i_^c^, µM)
EtOAc	>500 ppm	8.4 ± 0.8	NT ^d^
70% Ethanol	78.4 ± 2.4 ppm	74.2 ± 1.2	NT
H_2_O	284.7 ± 5.1 ppm	53.7 ± 1.6	NT
**1**	>100 µM	42.9 ± 1.4	NT
**2**	>100 µM	47.8 ± 1.1	NT
**3**	2.79 ± 0.2 µM	96.4 ± 0.7	Noncompetitive (3.1)
**4**	>100 µM	38.9 ± 1.9	NT
**5**	>100 µM	31.7 ± 1.3	NT
Allopurinol	2.28 ± 0.3 µM	>98	Competitive
Oxypurinol	28.4 ± 0.5 µM	87.6 ± 0.9	NT

^a^ All compounds were examined in a set of experiments repeated three times; ^b^ sample concentration was 200 ppm (µg/mL) for the extract and 100 µM for each compound; ^c^ values of inhibition constant; ^d^ NT is not tested; IC is inhibitory concentration.

**Table 2:** Spectral characteristics and contents (mg/g) of the five investigated compounds in the leaves of *T. sinensis*.

Peak	*t*_R_	*λ*_max_	Dried Leaves (mg/g) ^a^			[M − H]^−^ (*m/z*)	Molecular Formula	Identification ^b^
	(min)	(nm)	EtOAc	70% EtOH	H_2_O	(ESI-HRMS)	(ppm Error)	
**1**	5.90	254, 352	tr	0.69	0.71	609.1448	C_27_H_29_O_16_ (−1.3)	Quer-3-*O*-Rut
**2**	6.23	255, 353	tr	1.32	0.40	463.0893	C_21_H_19_O_12_ (3.5)	Quer -3-*O*-β-d-Glc
**3**	7.37	279	tr	1.34	0.25	939.1119	C_41_H_31_O_26_ (1.6)	PGG
**4**	7.79	255, 348	tr	7.12	2.05	447.0939	C_21_H_19_O_11_ (2.7)	Quer-3-*O*-α-l-Rham
**5**	9.56	263, 344	tr	1.46	0.41	431.0974	C_21_H_19_O_10_ (−0.9)	Kaem-3-*O*-α-l-Rham

^a^ All values are expressed as mean (*n* = 3); content expressed as mg of each compound equivalents per g of dry weight; ^b^ quer: quercetin; kaem: kaempferol; PGG: 1,2,3,4,6-penta-*O*-galloyl-β-d-glucopyranose; rut: rutinoside; glc: glucopyranoside; rham: rhamnopyranoside.

## Supplementary Materials

The following are available online: Figure S1–S5: ^1^H-NMR spectrum of compounds (**1**−**5**); Figure S6–S10: ^13^C-NMR spectrum of compounds (**1**−**5**); Figure S11–S15: Identification of compounds (**1**−**5**) by UPLC-qTof MS; Figure S16: Effects of compound **4** (40 mg/kg) on serum uric acid levels in PO-induced hyperuricemic rats.
