# Supplementary material for: Effects of Toona sinensis Leaf Extract and Its Chemical Constituents on Xanthine Oxidase Activity and Serum Uric Acid Levels in Potassium Oxonate-Induced Hyperuricemic Rats
Source: Molecules. 2018 Dec 9;23(12):3254. doi: 10.3390/molecules23123254 (PMC6321014; doi:10.3390/molecules23123254)
Supplement: Supplementary file 1 [file molecules-23-03254-s001.pdf]

Supplementary Materials

Effects of *Toona sinensis* leaf extract and its chemical constituents on xanthine oxidase activity and serum uric acid levels in potassium oxonate-induced hyperuricemic rats

Heung Joo Yuk [1,†], Young-Sil Lee [1,†], Hyung Won Ryu [2], Seung-Hyung Kim [3] and Dong-Seon Kim [1,*]

Title page ………………………………………………………………………………………. 1

Figure S1−5. ^{1}H-NMR spectrum of compounds (**1**−**5**) ……………………………………………………………... 2−6

Figure S6−10. ^{13}C-NMR spectrum of compounds (**1**−**5**) …………………………………………………………… 7−11

Figure S11−15. Identification of compounds (**1**−**5**) by UPLC-qTof MS ………………………………………………. 12−16

Figure S16. Effects of compound **4** (40 mg/kg) on serum uric acid levels in PO-induced hyperuricemic rats ………………..……... 17

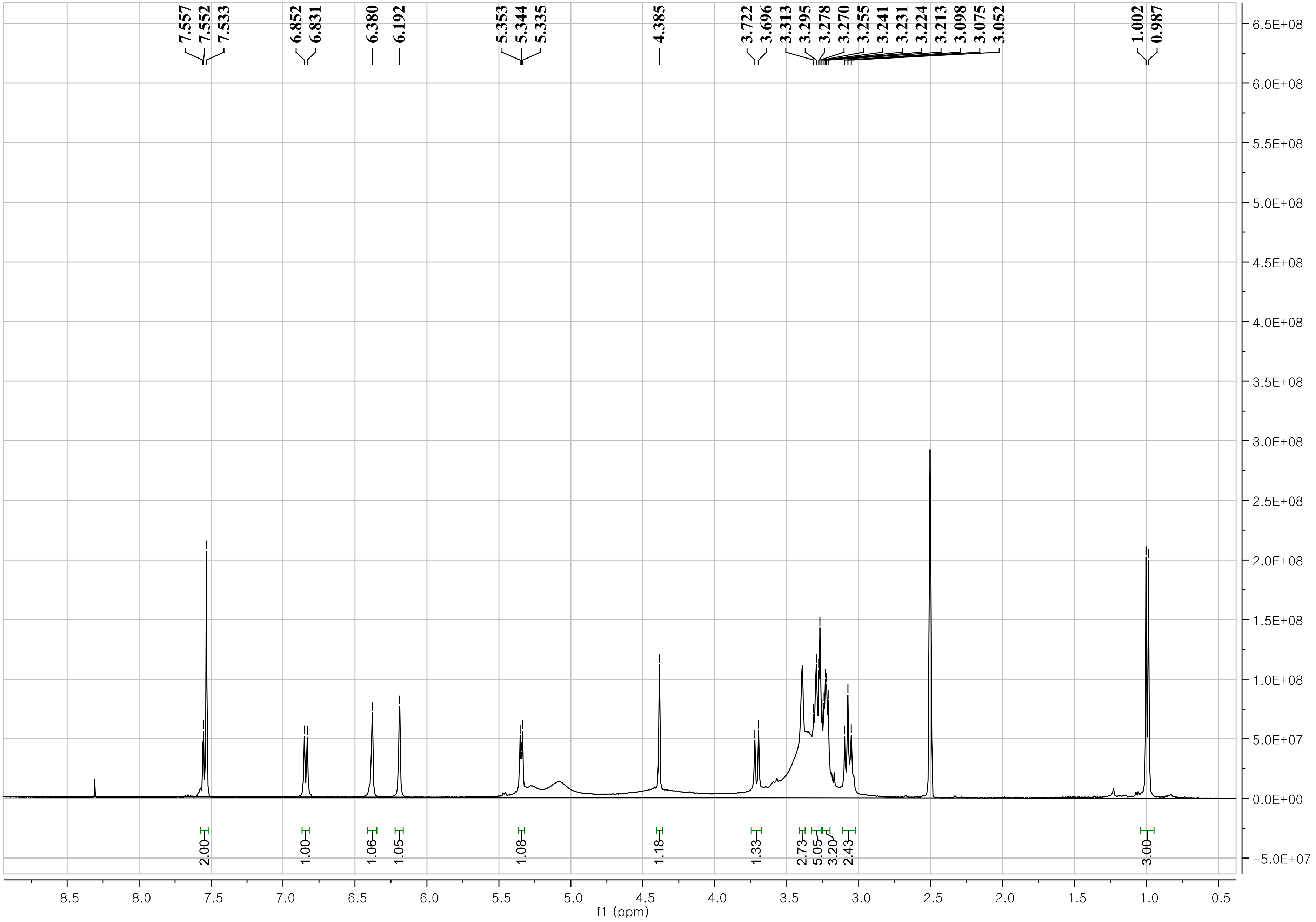

Figure S1. ^{1}H-NMR spectrum of compound **1** (400 MHz, DMSO-d_6).

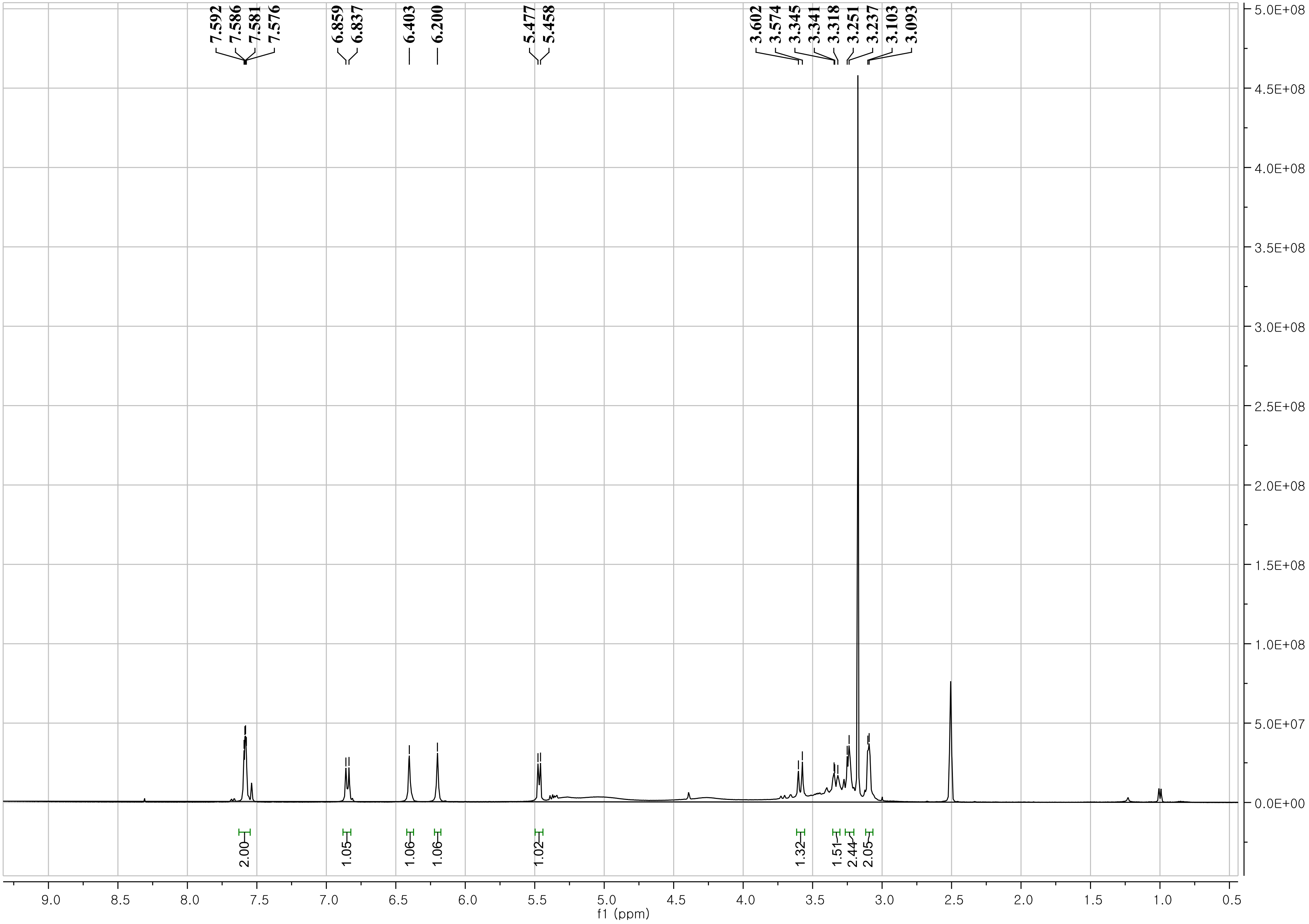

Figure S2. ^{1}H-NMR spectrum of compound **2** (400 MHz, DMSO-d_6).

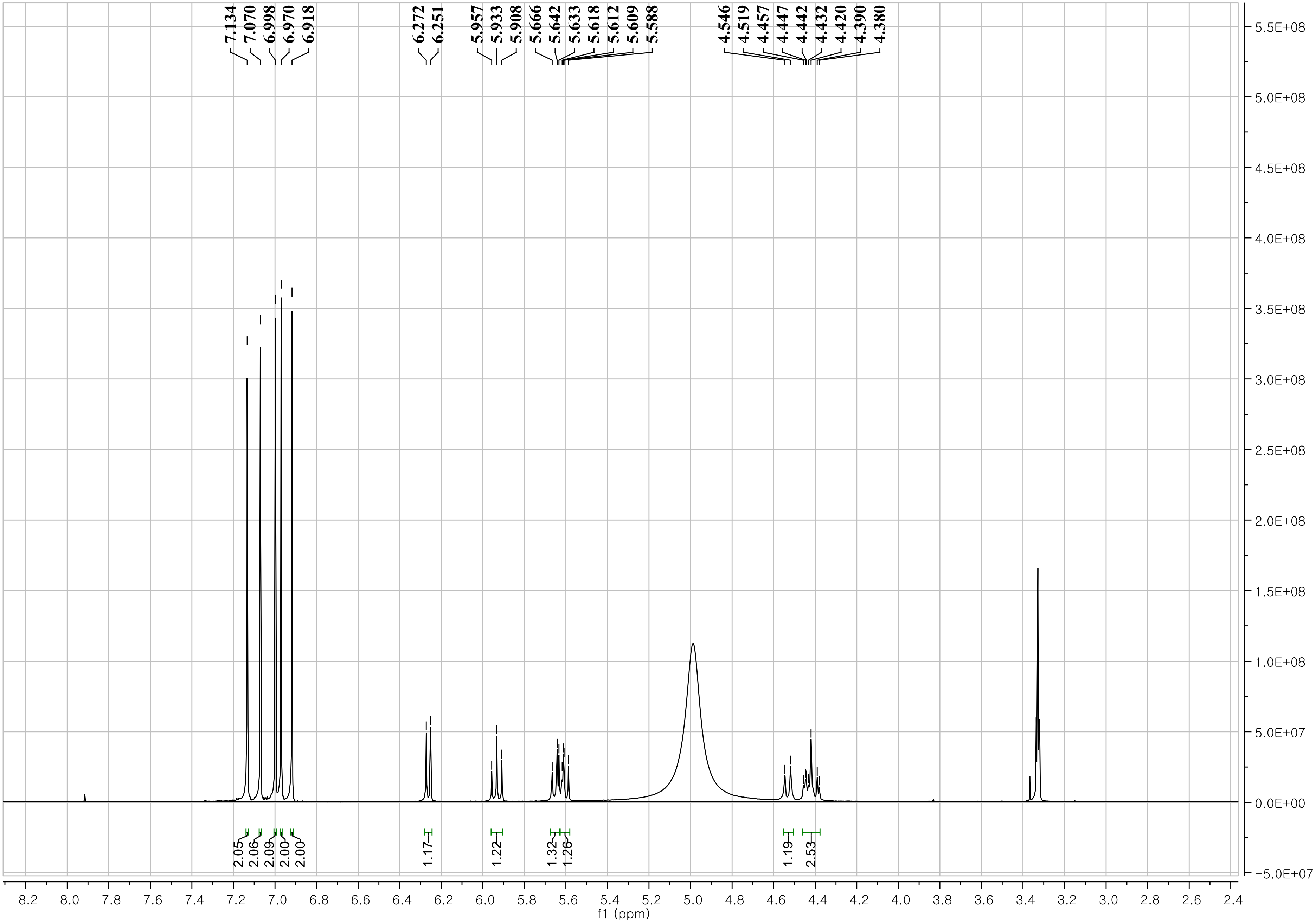

Figure S3. ^{1}H-NMR spectrum of compound **3** (400 MHz, CD_3OD).

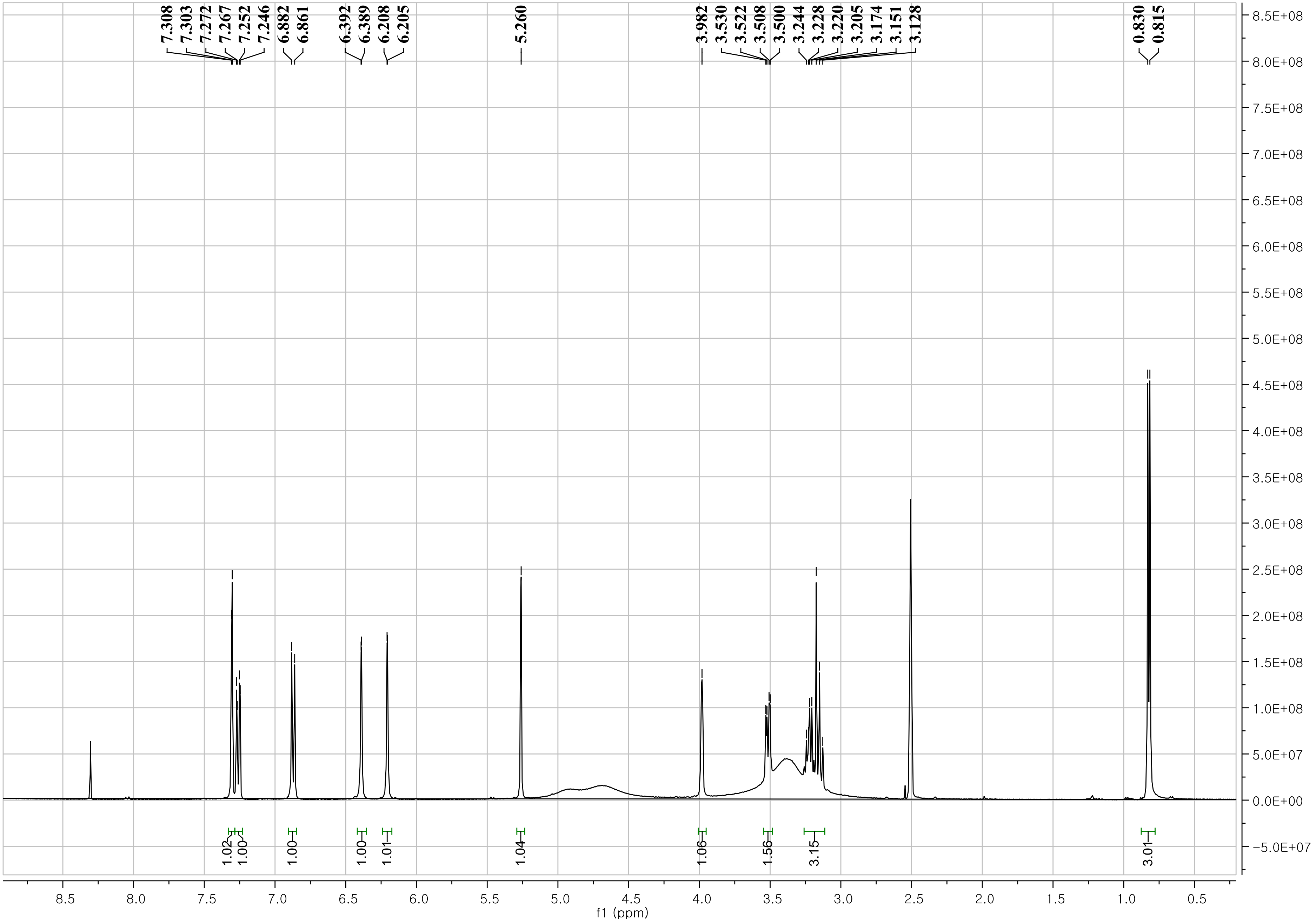

Figure S4. ^{1}H-NMR spectrum of compound **4** (400 MHz, DMSO-d_6).

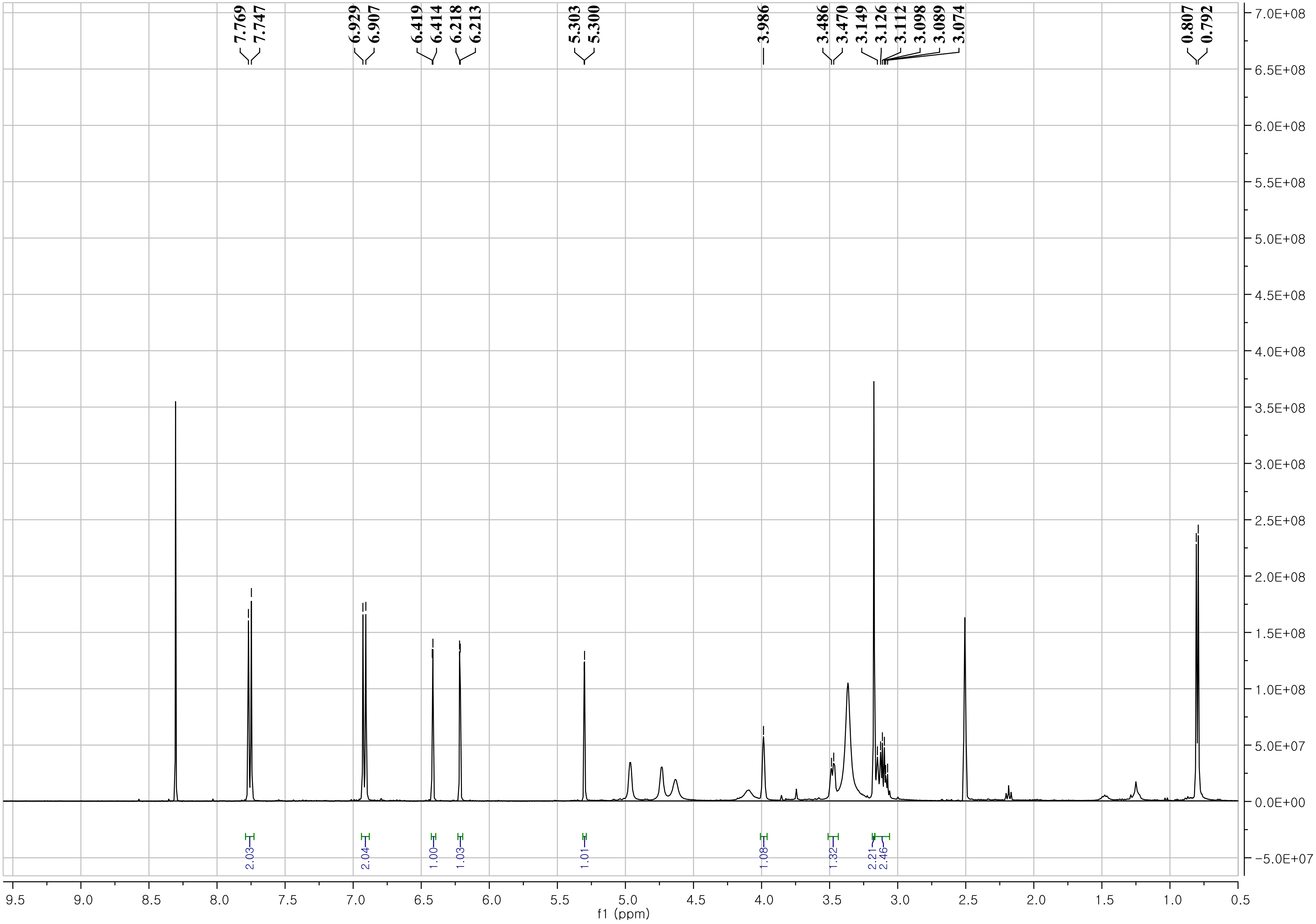

Figure S5. ^{1}H-NMR spectrum of compound **5** (400 MHz, DMSO-d_6).

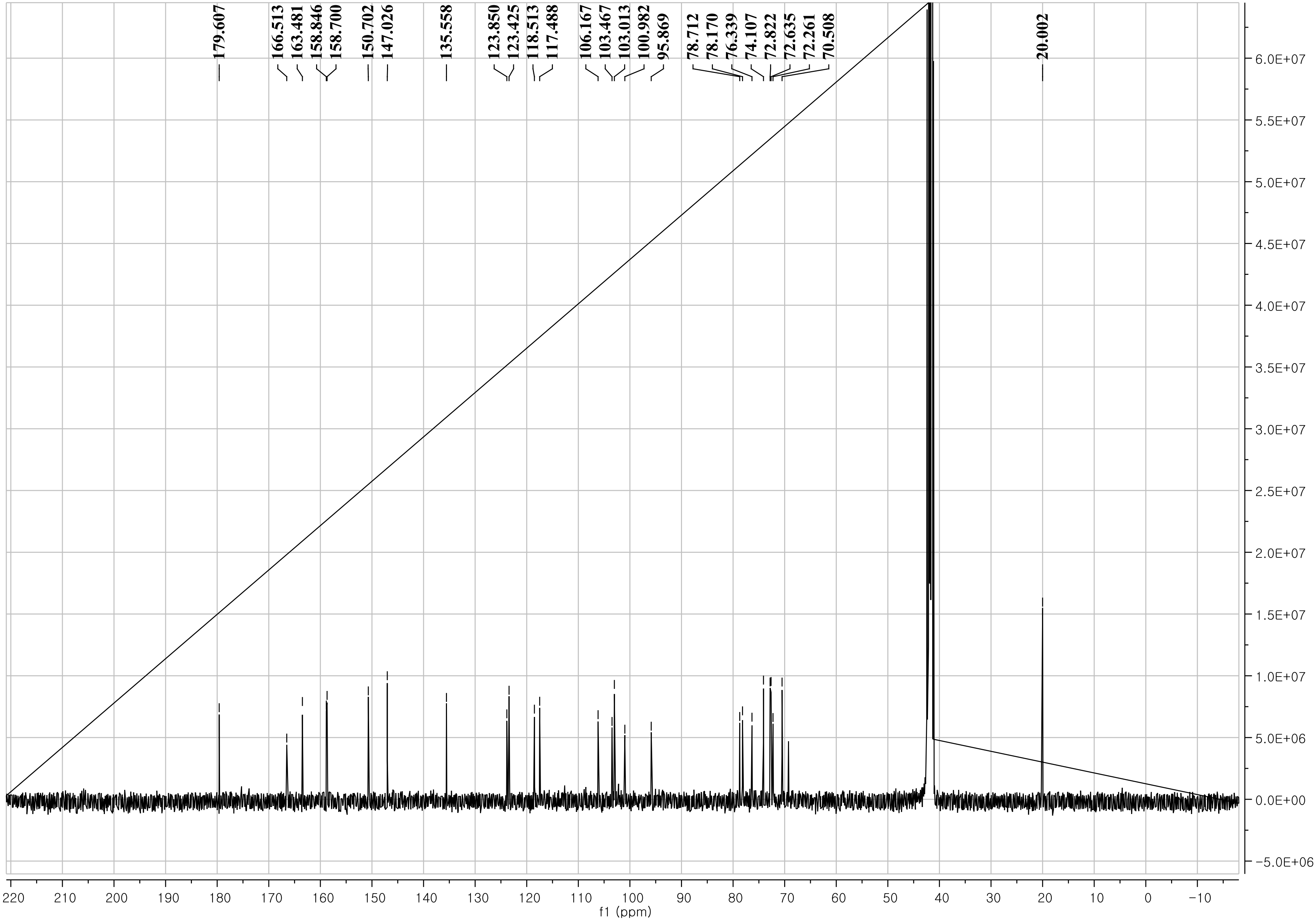

Figure S6. ^{13}C-NMR spectrum of compound **1** (100 MHz, DMSO-d_6).

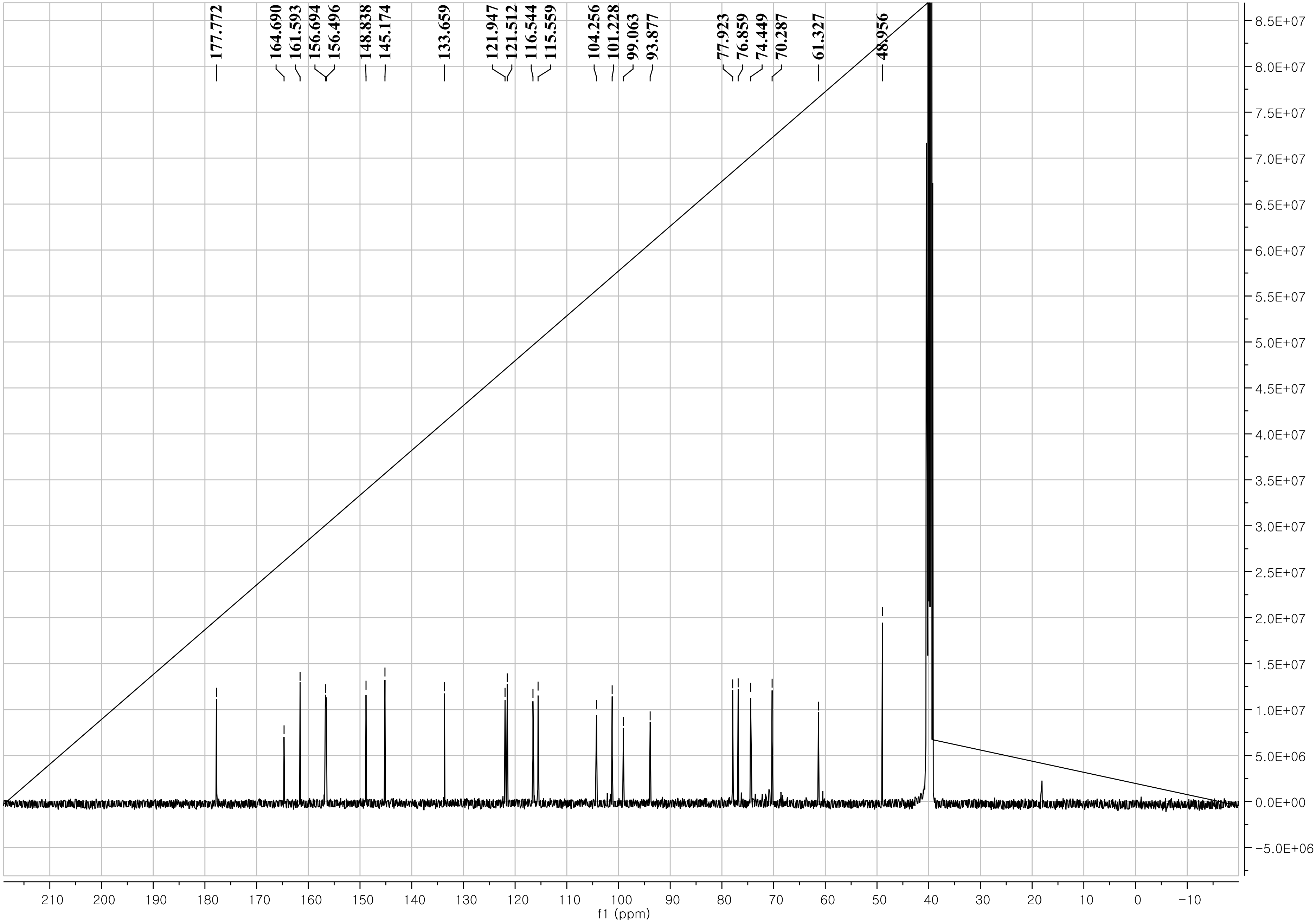

Figure S7. ^{13}C-NMR spectrum of compound **2** (100 MHz, DMSO-d_6).

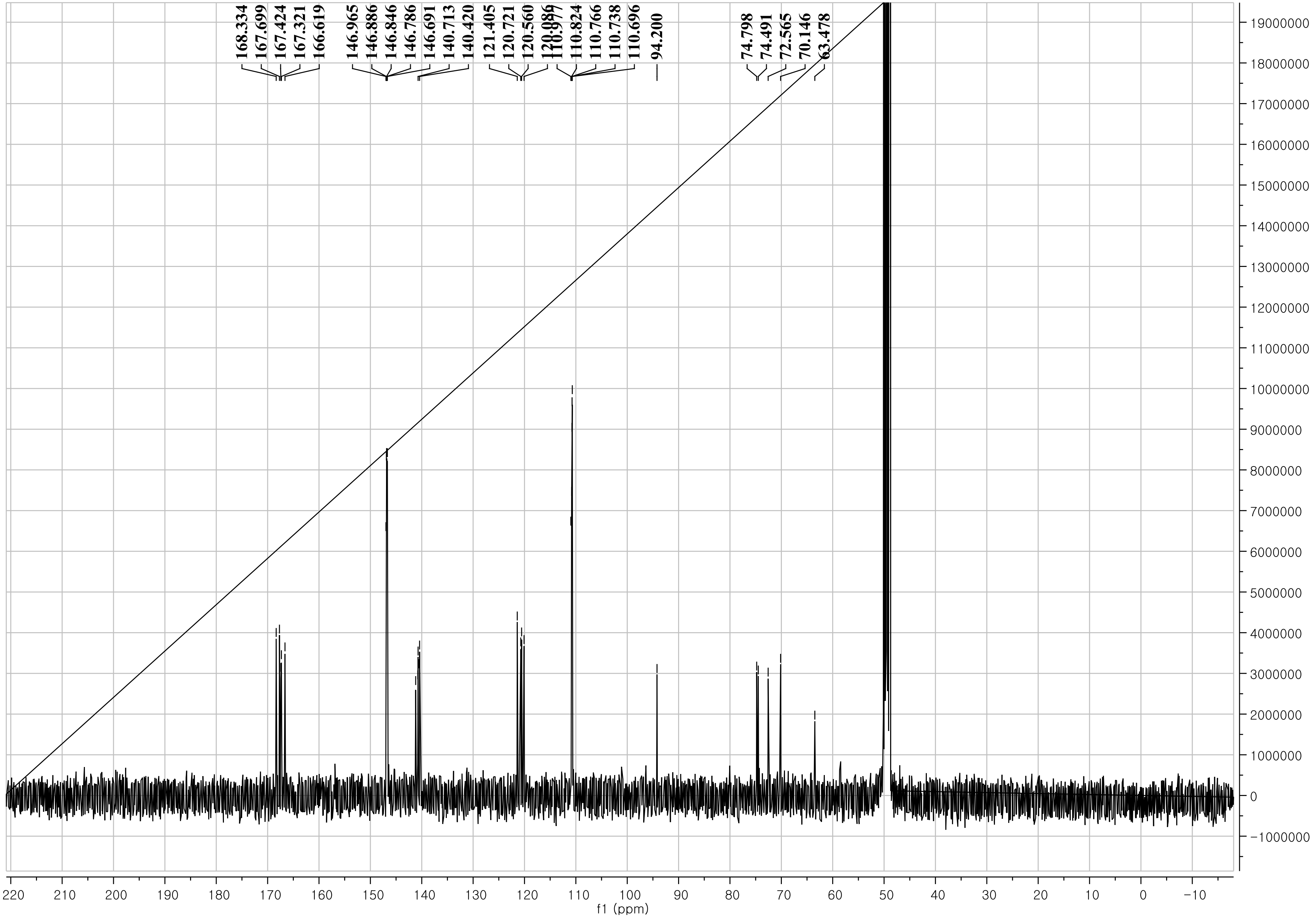

Figure S8. ^{13}C-NMR spectrum of compound **3** (100 MHz, CD_3OD).

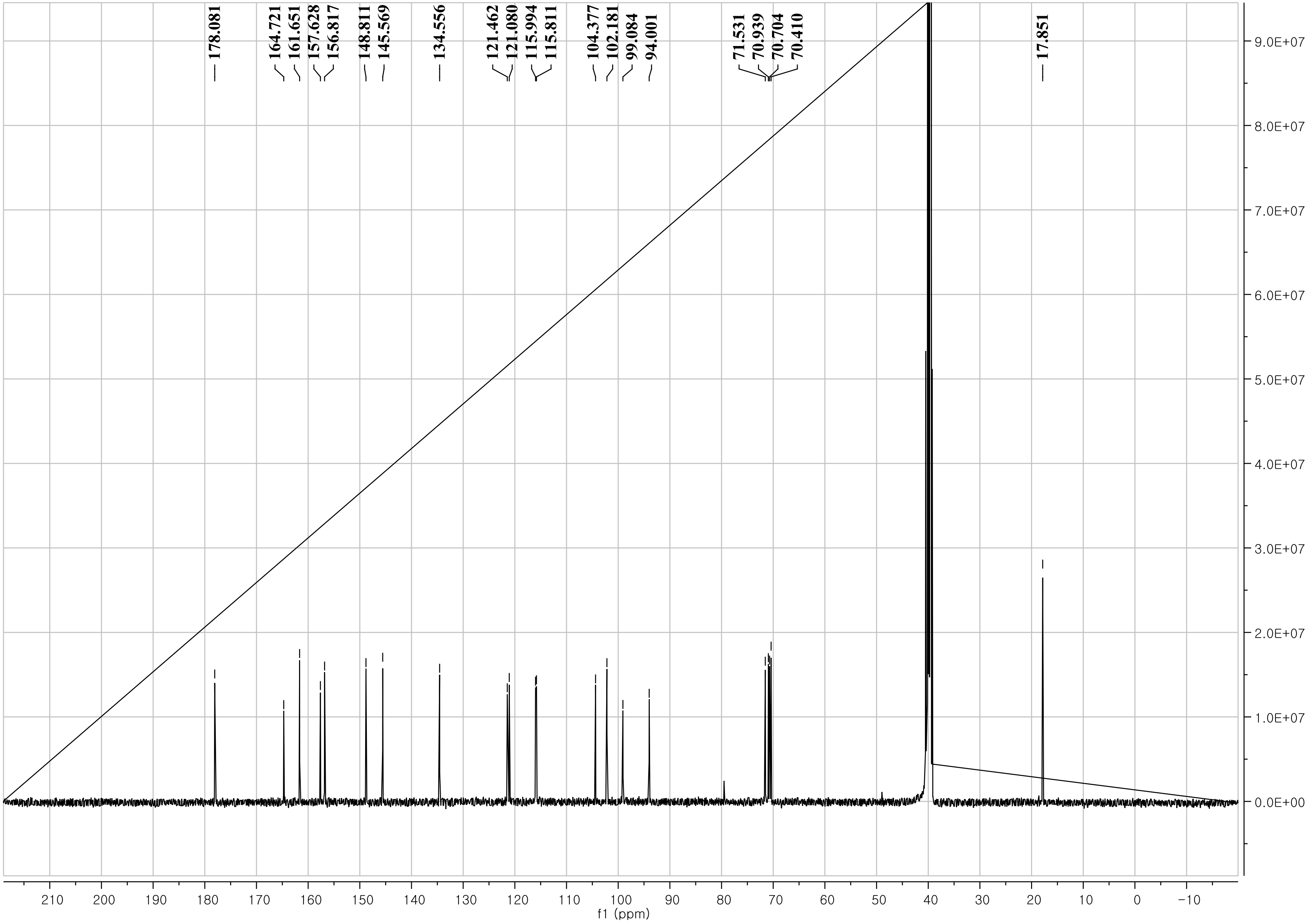

Figure S9. ^{13}C-NMR spectrum of compound **4** (100 MHz, DMSO-d_6).

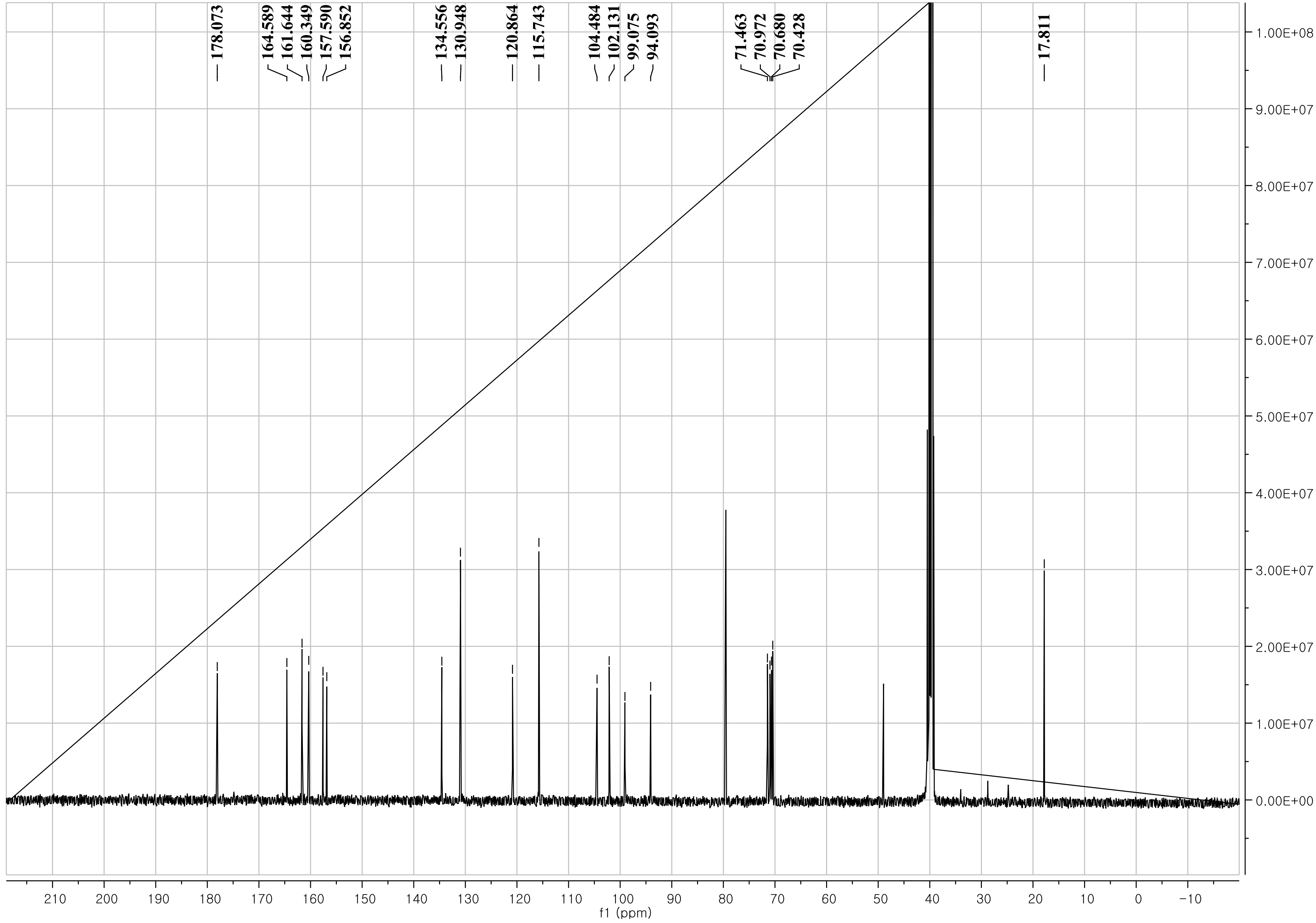

Figure S10. ^{13}C-NMR spectrum of compound **5** (100 MHz, DMSO-d_6).

Compound 1

[UV spectrum]

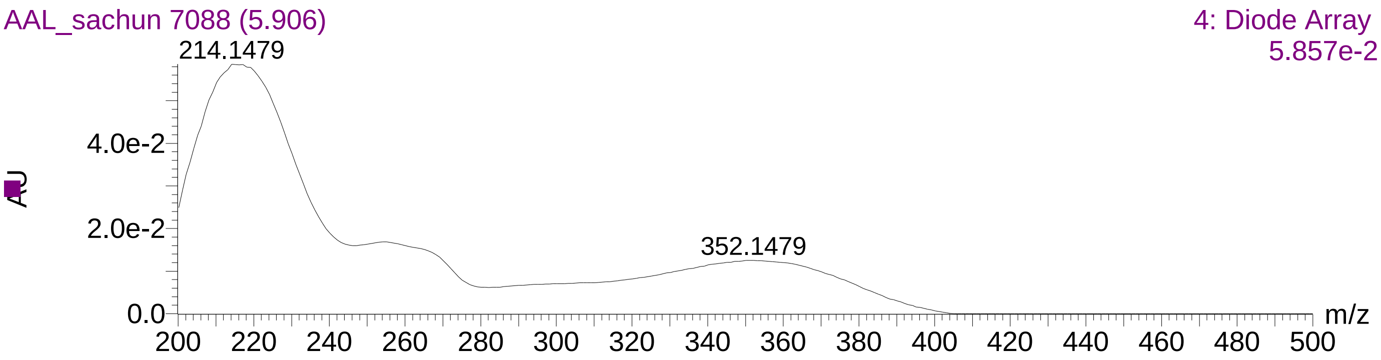

[MS Chromatogram]

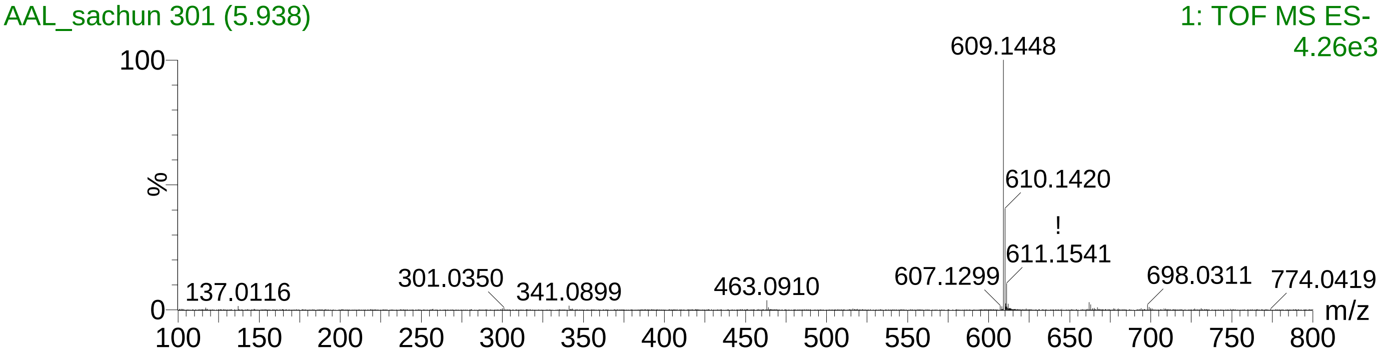

[MS^2 Chromatogram]

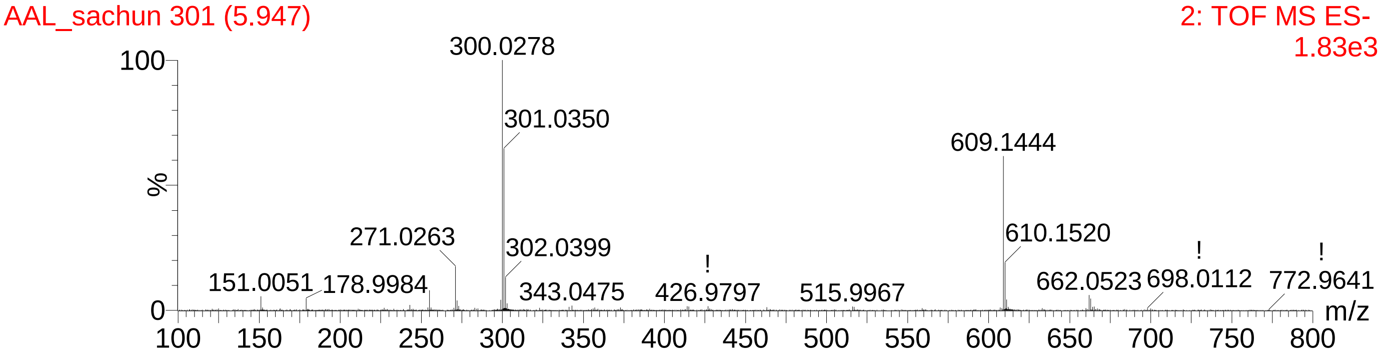

[Elemental Composition: HR-ESI/MS]

Single Mass Analysis

Tolerance = 5.0 PPM / DBE: min = -1.5, max = 50.0

Element prediction: Off

Number of isotope peaks used for i-FIT = 3

Monoisotopic Mass, Even Electron Ions

178 formula(e) evaluated with 1 results within limits (up to 50 best isotopic matches for each mass)

Elements Used:

Mass	Calc. Mass	mDa	PPM	DBE	Formula	i-...	i-FIT Norm	Fit Conf %	C	H	O
609.1448	609.1456	-0.8	-1.3	13.5	C27 H29 O16	59.6	n/a	n/a	27	29	16

Figure S11. Identification of compound **1** by UPLC-qTof MS.

Compound 2

[UV spectrum]

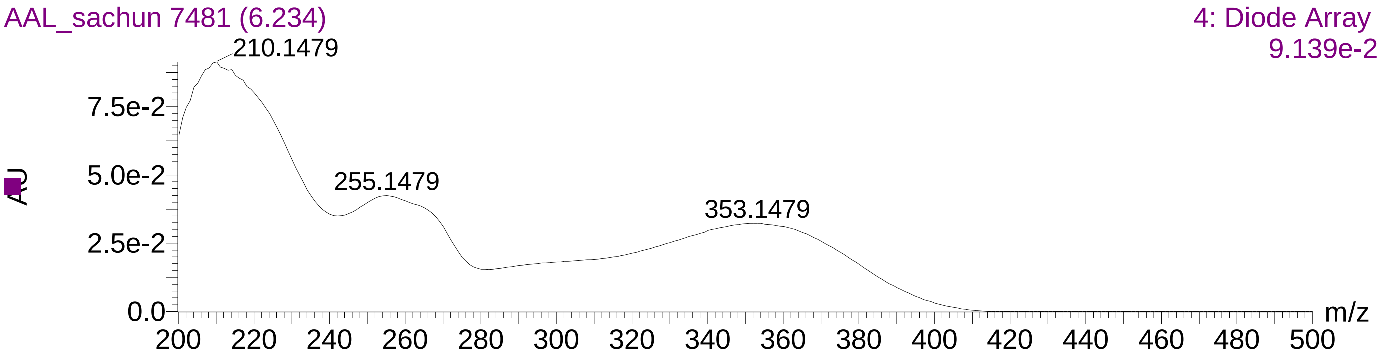

[MS Chromatogram]

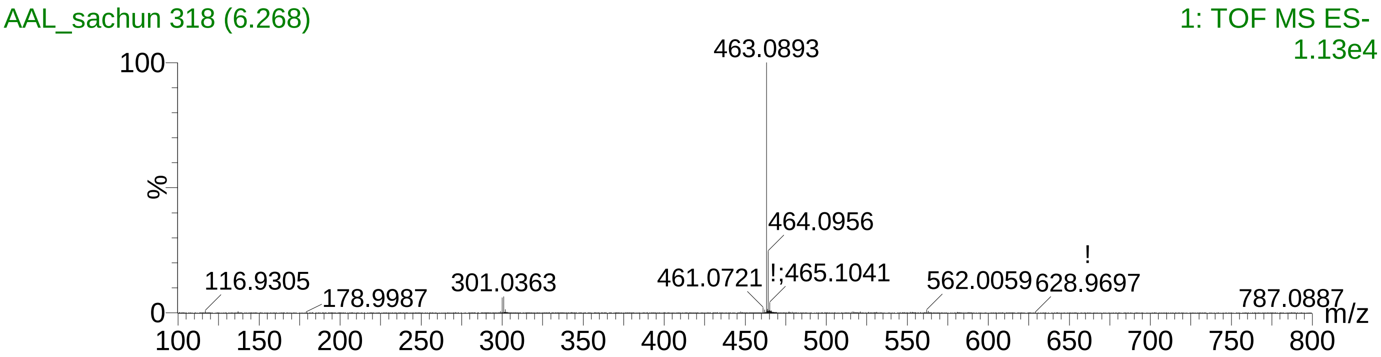

[MS^2 Chromatogram]

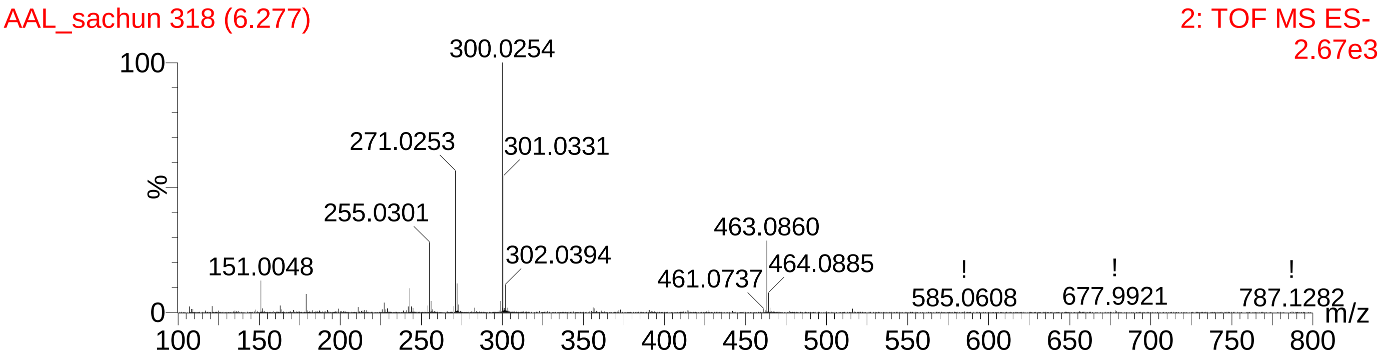

[Elemental Composition: HR-ESI/MS]

Single Mass Analysis

Tolerance = 5.0 PPM / DBE: min = -1.5, max = 50.0

Element prediction: Off

Number of isotope peaks used for i-FIT = 3

Monoisotopic Mass, Even Electron Ions

114 formula(e) evaluated with 1 results within limits (up to 50 best isotopic matches for each mass)

Elements Used:

Mass	Calc. Mass	mDa	PPM	DBE	Formula	i-FIT	i-FIT Norm	Fit Conf %	C	H	O
463.0893	463.0877	1.6	3.5	12.5	C21 H19 O12	159.9	n/a	n/a	21	19	12

Figure S12. Identification of compound **2** by UPLC-qTof MS.

Compound 3

[UV spectrum]

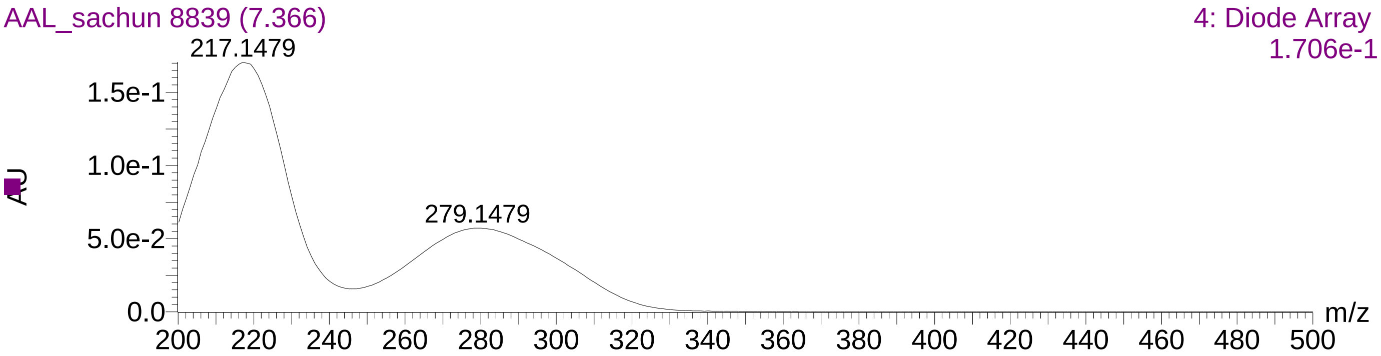

[MS Chromatogram]

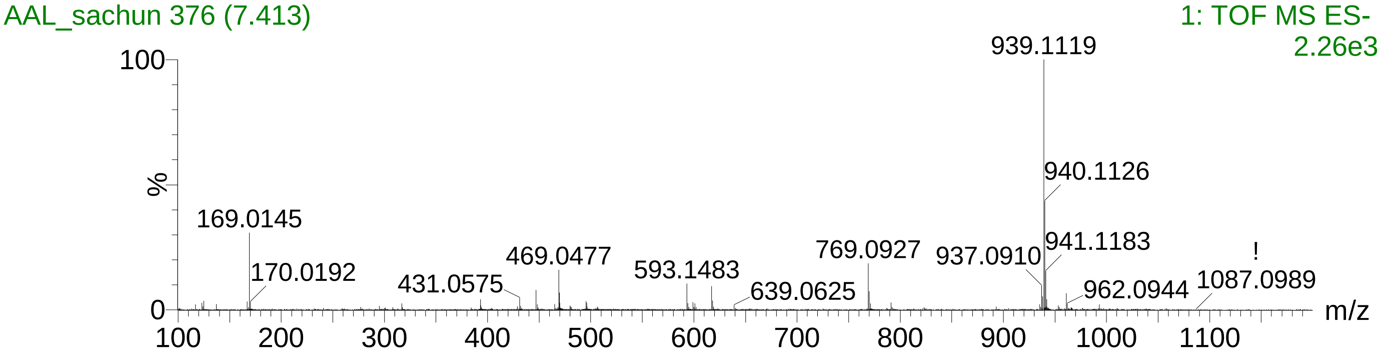

[MS^2 Chromatogram]

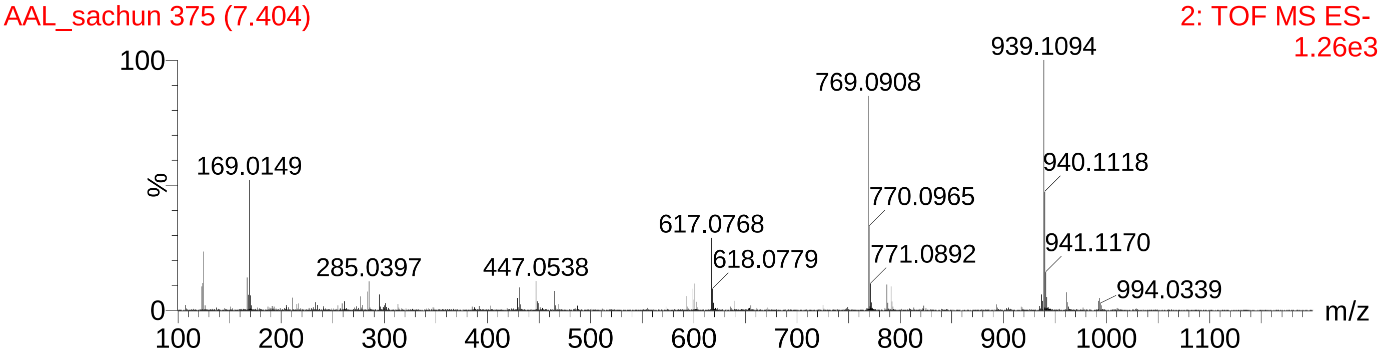

[Elemental Composition: HR-ESI/MS]

Single Mass Analysis

Tolerance = 5.0 PPM / DBE: min = -1.5, max = 50.0

Element prediction: Off

Number of isotope peaks used for i-FIT = 3

Monoisotopic Mass, Even Electron Ions

399 formula(e) evaluated with 3 results within limits (up to 50 best isotopic matches for each mass)

Elements Used:

Mass	Calc, Mass	mDa	PPM	DBE	Formula	i-...	i-FIT Norm	Fit Conf %	C	H	O
939,1119	939,1104	1,5	1,6	26,5	C41 H31 O26	43,2	0,007	99,31	41	31	26
	939,1162	-4,3	-4,6	17,5	C34 H35 O31	48,2	5,017	0,66	34	35	31
	939,1139	-2,0	-2,1	48,5	C59 H23 O13	51,3	8,097	0,03	59	23	13

Figure S13. Identification of compound **3** by UPLC-qTof MS.

Compound 4

[UV spectrum]

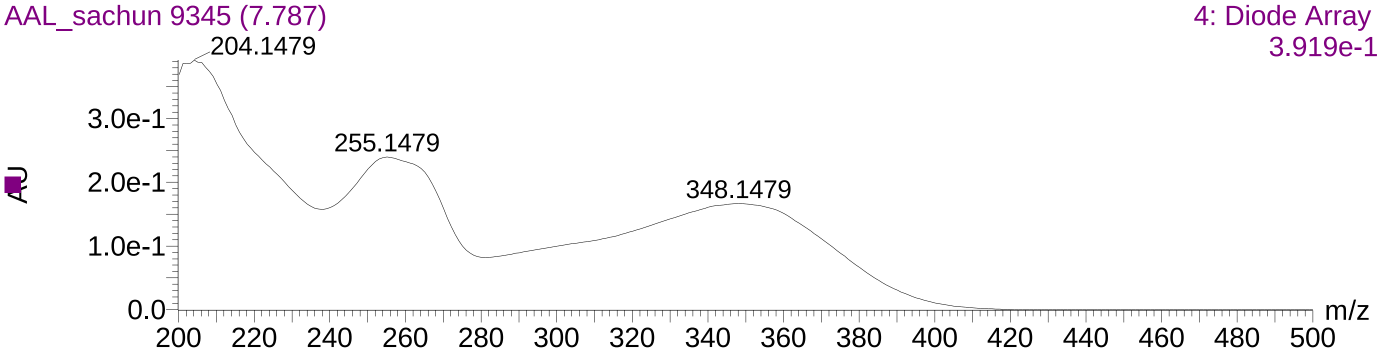

[MS Chromatogram]

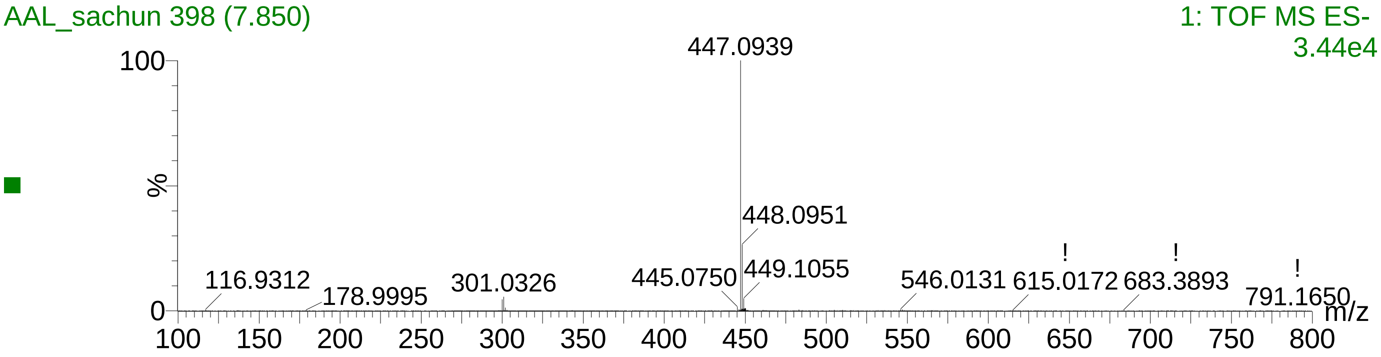

[MS² Chromatogram]

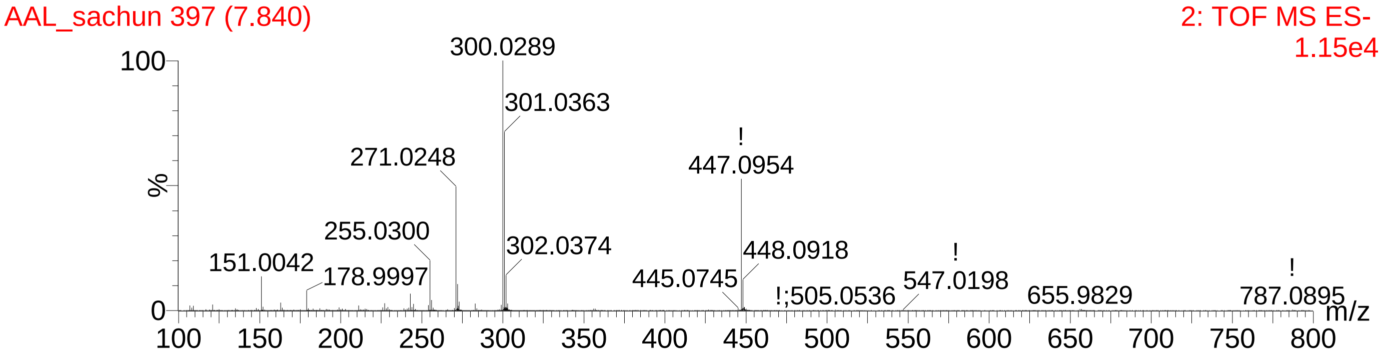

[Elemental Composition: HR-ESI/MS]

Single Mass Analysis

Tolerance = 5.0 PPM / DBE: min = -1.5, max = 50.0

Element prediction: Off

Number of isotope peaks used for i-FIT = 3

Monoisotopic Mass, Even Electron Ions

108 formula(e) evaluated with 1 results within limits (up to 50 best isotopic matches for each mass)

Elements Used:

Mass	Calc. Mass	mDa	PPM	DBE	Formula	i-FIT	i-FIT Norm	Fit Conf %	C	H	O
447.0939	447.0927	1.2	2.7	12.5	C21 H19 O11	188.1	n/a	n/a	21	19	11

Figure S14. Identification of compound **4** by UPLC-qTof MS.

Compound 5

[UV spectrum]

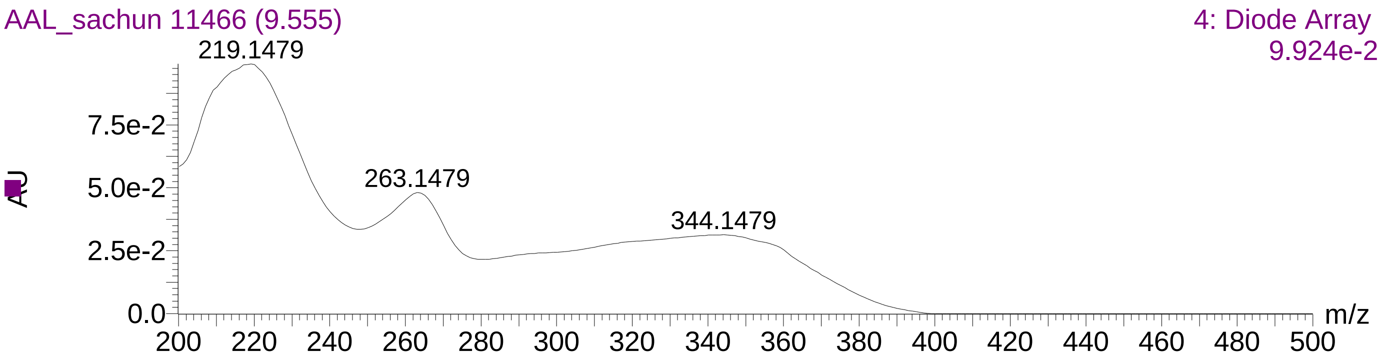

[MS Chromatogram]

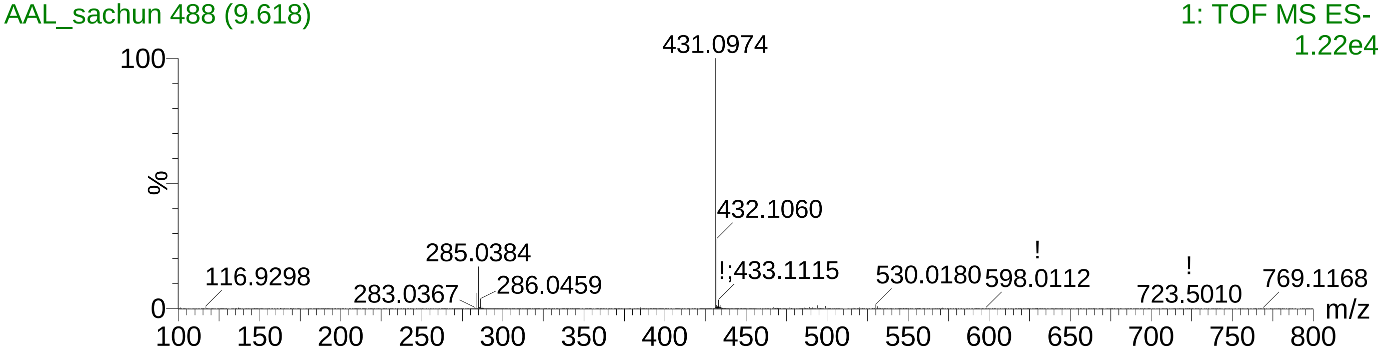

[MS² Chromatogram]

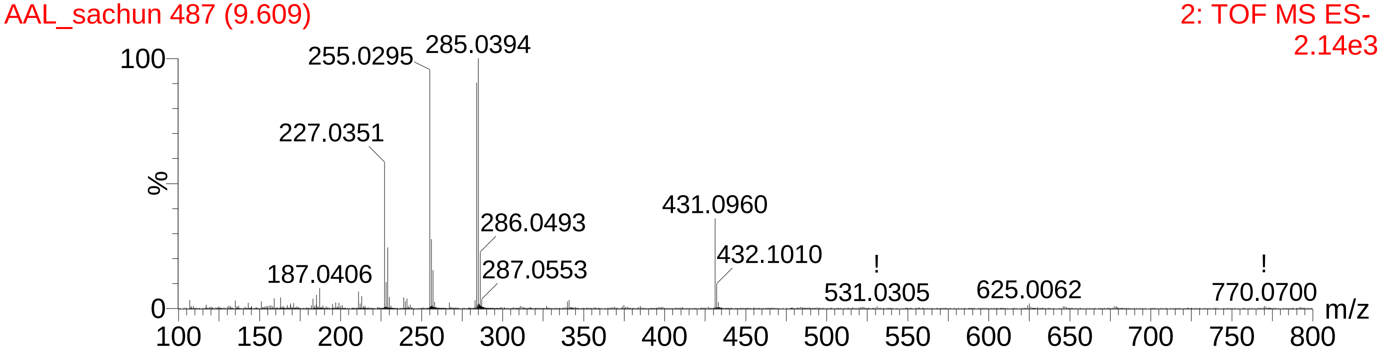

[Elemental Composition: HR-ESI/MS]

Single Mass Analysis
Tolerance = 5.0 PPM / DBE: min = -1.5, max = 50.0
Element prediction: Off
Number of isotope peaks used for i-FIT = 3
Monoisotopic Mass, Even Electron Ions
102 formula(e) evaluated with 1 results within limits (up to 50 best isotopic matches for each mass)
Elements Used:

Mass	Calc. Mass	mDa	PPM	DBE	Formula	i-FIT	i-FIT Norm	Fit Conf %	C	H	O
431.0974	431.0978	-0.4	-0.9	12.5	C21 H19 O10	164.4	n/a	n/a	21	19	10

Figure S15. Identification of compound **5** by UPLC-qTof MS.

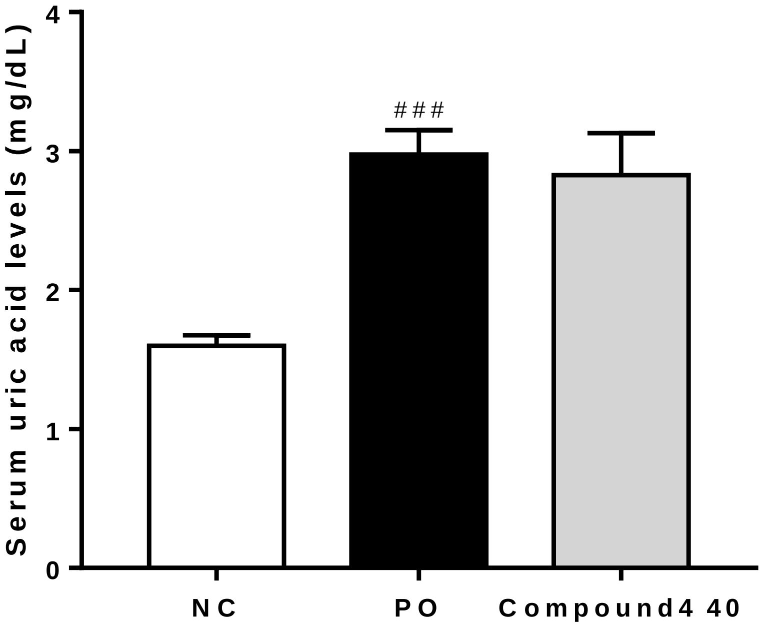

Figure S16. Effects of compound **4** (40 mg/kg) on serum uric acid levels in PO-induced hyperuricemic rats. NC, normal control group; PO, potassium oxonate-induced hyperuricemia group. Data are expressed as the mean ± SEM (*n*=6). $^{###}p<0.001$ vs. the NC group; $^{*}p<0.05$ and $^{***}p<0.005$ vs. the PO group.
